# NGF Accelerates Cutaneous Wound Healing by Promoting the Migration of Dermal Fibroblasts via the PI3K/Akt-Rac1-JNK and ERK Pathways

**DOI:** 10.1155/2014/547187

**Published:** 2014-05-21

**Authors:** Ji-Cai Chen, Bei-Bei Lin, Hou-Wen Hu, Cai Lin, Wen-Yang Jin, Fa-Biao Zhang, Yan-An Zhu, Cai-Jiao Lu, Xiao-Jie Wei, Rui-Jie Chen

**Affiliations:** ^1^Department of Gastrointestinal Surgery, The First Affiliate Hospital, Wenzhou Medical University, Wenzhou 325035, China; ^2^School of Pharmacy, Wenzhou Medical University, Wenzhou 325035, China; ^3^Wound Treatment Center, The First Affiliate Hospital, Wenzhou Medical University, Wenzhou 325000, China; ^4^Emergency Department, Taizhou Hospital, Wenzhou Medical University, Taizhou 318000, China; ^5^Translation Medicine Research Center, Cixi People's Hospital, Wenzhou Medical University, Ningbo 315300, China; ^6^Departmant of Pharmacy, The Second Affiliate Hospital, Wenzhou Medical University, Wenzhou 325000, China

## Abstract

As a well-known neurotrophic factor, nerve growth factor (NGF) has also been extensively recognized for its acceleration of healing in cutaneous wounds in both animal models and randomized clinical trials. However, the underlying mechanisms accounting for the therapeutic effect of NGF on skin wounds are not fully understood. NGF treatment significantly accelerated the rate of wound healing by promoting wound reepithelialization, the formation of granulation tissue, and collagen production. To explore the possible mechanisms of this process, the expression levels of CD68, VEGF, PCNA, and TGF-*β*1 in wounds were detected by immunohistochemical staining. The levels of these proteins were all significantly raised in NGF-treated wounds compared to untreated controls. NGF also significantly promoted the migration, but not the proliferation, of dermal fibroblasts. NGF induced a remarkable increase in the activity of PI3K/Akt, JNK, ERK, and Rac1, and blockade with their specific inhibitors significantly impaired the NGF-induced migration. In conclusion, NGF significantly accelerated the healing of skin excisional wounds in rats and the fibroblast migration induced by NGF may contribute to this healing process. The activation of PI3K/Akt, Rac1, JNK, and ERK were all involved in the regulation of NGF-induced fibroblast migration.

## 1. Introduction

The skin is the largest organ in the body, covering the entire external surface, and it has many different functions. The skin primarily acts as a protective physical barrier between the host and the external environment against numerous insults, and it serves to prevent excessive loss of body water while also possessing other critical functions including immune surveillance, sensory detection, and self-healing. The breakdown of skin integrity because of injury or illness such as diabetes, pressure, or venous stasis results in substantial physiologic imbalance and renders a patient vulnerable to a number of pathologic conditions, such as infection, fluid loss, and electrolyte imbalance [1]. Therefore, the efficient and complete healing of cutaneous wounds is undoubtedly critical.

The healing of skin wounds is a complex process involving a series of sequential and overlapping phases such as clotting, inflammatory infiltration, reepithelialization, and the formation of granulation tissue, followed by tissue remodeling and wound contraction [2]. This process requires the collaboration of a variety of tissue and cell types, including inflammatory cells, fibroblasts, keratinocytes, endothelial cells, and macrophages. These cells are tightly regulated by cytokines, growth factors, and extracellular matrix (ECM) molecules [3]. Inflammatory cells, keratinocytes, and fibroblasts in the wound area and border produce and release a variety of growth factors such as platelet-derived growth factor (PDGF), epidermal growth factor (EGF), fibroblast growth factor (FGF), transforming growth factor (TGF), and nerve growth factor (NGF) [4]. Local or topical application of exogenous growth factors and cytokines has also been reported to accelerate the repair of acute and chronic wounds [5].

NGF is the first isolated and best-characterized member of a neurotrophin family. NGF exerts a crucial role in survival, differentiation, and function of peripheral sensory and sympathetic nerves and brain neurons of mammals [6]. Additionally, accumulating evidence has shown that NGF plays a prominent role in promoting healing processes. NGF levels are significantly higher at wounded sites after skin punching than at uninjured control skin sites [7]. The removal of the submaxillary glands, tissues storing large amounts of NGF, substantially affects recovery of wound healing in mouse skin, and when purified NGF is topically applied to mice without submandibular glands, the rate of wound contraction is markedly accelerated [8]. The application of NGF to cutaneous wounds accelerated the rate of wound healing in both normal and healing-impaired diabetic mice [7]. Topical application of NGF also promotes skin ulcer healing in patients with pressure ulcers [9]. However, the regulatory mechanism underlying NGF-accelerated cutaneous wound healing is not yet completely understood. Thus, the underlying mechanism deserves further investigations.

Dermal fibroblasts play critical roles in all three phases of wound healing. After wounding, fibroblasts are attracted from the edge of the wound or from the bone marrow [10]. At the inflammation stage, fibroblasts produce a variety of chemokines [11]. At the stage of new tissue formation, fibroblasts are stimulated by macrophages and some differentiate into myofibroblasts. Fibroblasts interact with myofibroblasts to produce extracellular matrix, mainly in the form of collagen [12]. At the stage of tissue remodeling, most of the myofibroblasts, macrophages, and endothelial cells undergo apoptosis or exit from the wound, leaving a mass that contains few cells and consists mostly of collagen and other extracellular matrix proteins [1]. However, the effect of NGF on dermal fibroblasts is largely unknown.

To address these questions, the present study first examined the effect of topical NGF on wound healing in a rat skin excisional wound model and then further explored the possible mechanisms of wound-healing promoted by NGF. In light of the crucial role that dermal fibroblasts play in wound healing, we mainly focused on investigating the effect of NGF on dermal fibroblasts and underlying signal pathways.

## 2. Results

### 2.1. NGF Accelerated the Rate of Cutaneous Wound Healing

**Figure 1:**
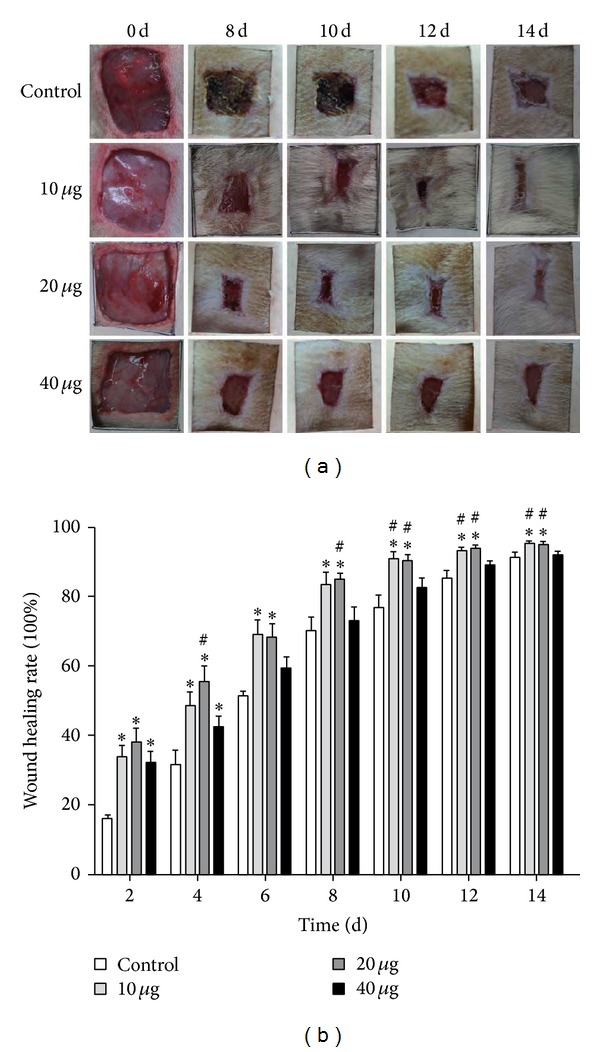
Wound closure after NGF treatment in rat. (a) Representative photographs of skin full-thickness excisional wounds in rat treated topically every other day with saline control or 10, 20, or 40 *μ*g/mL of NGF for 8, 10, 12, and 14 days after wounding. (b) The rate of wound healing after different concentrations of NGF treatment. *P < 0.05, compared to saline-treated control; ^#^ *P* < 0.05, compared to 40 *μ*g/mL NGF group; *n* = 7.

The healing of skin excisional wounds in rats was determined by the percentage of the wound surface covered by regenerating epidermis. NGF-treated wounds exhibited accelerated skin wound closure in Sprague-Dawley rats compared with saline-treated wounds (Figure 1(a)). The enhancement of wound healing appeared as early as at 2 days following treatment with the three different doses of NGF (Figure 1(b)). Although 10 *μ*g or 20 *μ*g of NGF treatment demonstrated an accelerated speed in wound closure, the group treated with 40 *μ*g NGF did not show a significant difference from the saline-treated control between 6 days of treatment and the end of the experiment. After treatment with 10 *μ*g or 20 *μ*g of NGF for 10 days, the cutaneous wound was nearly closed, while the saline-treated group still showed a much larger wound area. It seems that topical NGF treatment reaches its maximal effect in wound healing at 20 *μ*g topically per wound. Therefore, the 20 *μ*g NGF treatment was chosen for our later experiment.

### 2.2. NGF Promoted Reepithelialization, Granulation Tissue Formation, and Collagen Production

**Figure 2:**
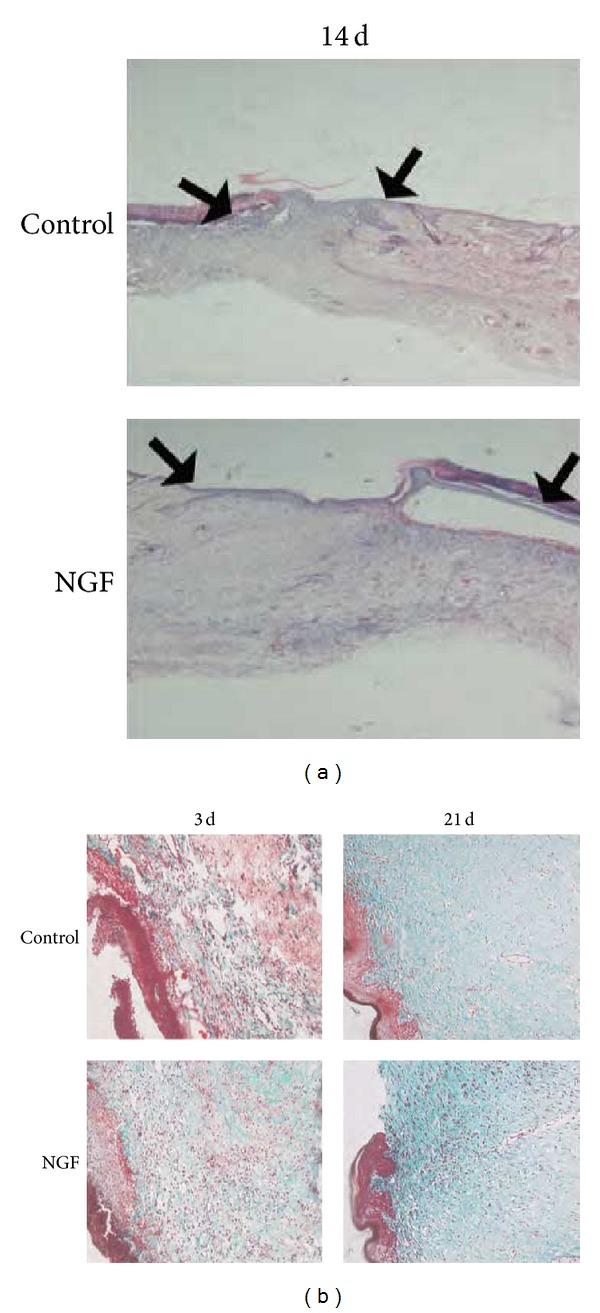
Histological structure of wounded skin sections. (a) Hematoxylin and eosin stainingof wound healing at 14 days after wounding (×40). As compared with saline-treated control, epithelial crawling (the distance between two black arrows as indicated) in NGF-treated wounds advanced more rapidly, and more granulation tissue was found in the NGF-treated wound. (b) Masson's trichrome staining of the wounded skin sections with saline or NGF treatment for 3 and 21 days after wounding (×200). There is a mild increase of collagen content (green) in NGF-treated wound tissue compared with saline-treated controls at different times after wounding.

Excisional skin wounds are healed through lateral migration of keratinocytes, called reepithelialization, followed by inward migration of dermal cells. The rate of wound closure was well matched to the histological observations in wounds by hematoxylin and eosin staining (Figure 2(a)). The NGF-treated wounds exhibited substantially more reepithelialization than the saline-treated wounds. Longer reepithelialized tongues (ReT) could be clearly visualized after 14 days of 20 *μ*g NGF treatment compared to saline-treated controls (Figure 2(a)). In addition, the granulation tissue formed in NGF-treated wounds appeared to be thicker and larger than saline-treated control. The sections stained with Masson's trichrome were shown in Figure 2(b). There was a mild increase in collagen production in NGF-treated wounds compared with the saline-treated controls. Although collagen staining was light and unevenly distributed, the collagen content in NGF-treated wounds was higher than in saline-treated controls at 3 days after wounding. The collagen in NGF-treated wounds at 21 days after wounding was more mature than in saline-treated controls.

### 2.3. NGF Enhanced the Expression of TGF-*β*1 and PCNA in Wound

**Figure 3:**
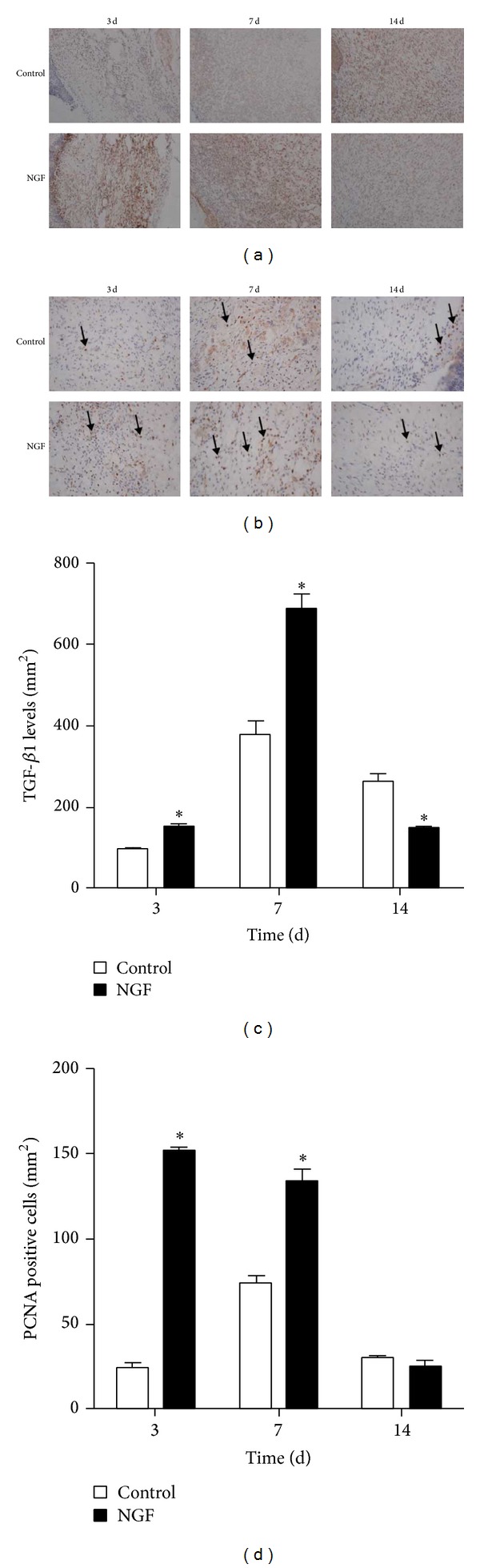
NGF administration increases the level of TGF-*β*1- and PCNA-positive cells in skin wounds. Immunohistochemical staining for (a) TGF-*β*1 and (b) PCNA was performed at the indicated day after wounding (×200). (c) The optical density of TGF-*β*1 analyzed with Image J software. (d) The number of cells positive for PCNA staining. *P < 0.05, compared to control group.

It has been extensively reported that collagen synthesis is modulated by the profibrotic cytokine transforming growth factor *β* (TGF-*β*). We therefore investigated the expression of TGF-*β*1 in NGF-treated and saline-treated wounds. As shown in Figures 3(a) and 3(c), the expression of TGF-*β*1 in NGF-treated wounds was significantly increased at 3 days and 7 days after wounding compared to saline-treated controls. However, the expression of TGF-*β*1 in the NGF-treated wound was significantly decreased at 14 days after wounding. To explore the effect of NGF treatment on cell proliferation, the expression of PCNA, a marker of cell proliferation, in the wound tissues was detected by immunohistochemical staining using an anti-PCNA antibody. As shown in Figures 3(b) and 3(d), the expression of PCNA was significantly induced in NGF-treated wounds compared with the saline-treated control at the early time points, 3 days and 7 days after wounding. However, there was no significant difference in PCNA expression between NGF-treated and saline-treated wounds at 14 days after wounding. These data suggest that NGF can promote cell proliferation and collagen production.

### 2.4. NGF Upregulated the Levels of CD68 and VEGF in Wounds

**Figure 4:**
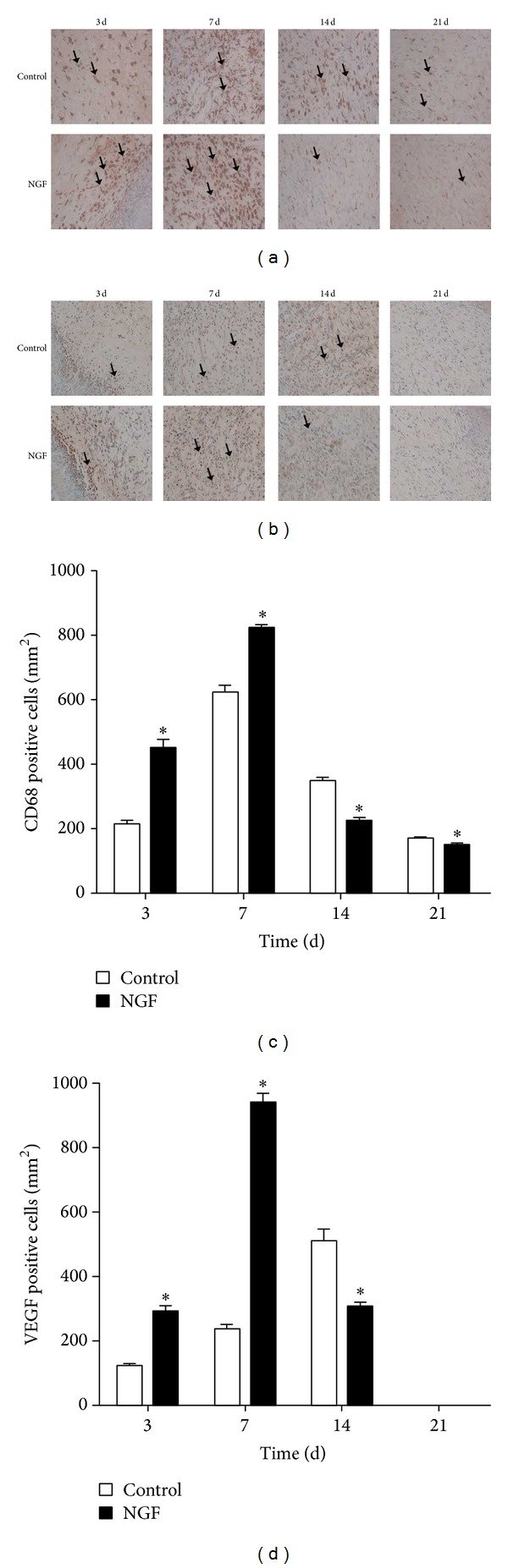
The expression of CD68 and VEGF was increased following NGF treatment. Immunohistochemical staining for CD68 (a) and VEGF (b) was performed at the indicated day after wounding (×200). ((c) and (d)) The number of cells positive for CD68 or VEGF in wounds. *P < 0.05, compared to control group.

The inflammatory response is instrumental for supplying the growth factor and cytokine signals that orchestrate the cell and tissue movements necessary for repair during wound healing. The expression of CD68, a marker for macrophages, was detected by immunohistochemical staining. As shown in Figures 4(a) and 4(c), there were more cells positive for CD68 in NGF-treated wounds than in saline-treated controls at 3 days and 7 days after wounding. However, the level of CD68 expression in NGF-treated wounds was lower than in saline-treated controls at 14 days and 21 days after wounding. These results suggested that NGF was involved in the early stage of inflammation by recruiting inflammatory cells such as macrophages. The expression of VEGF, a proangiogenic growth factor, in NGF-treated wounds was investigated by immunohistochemical staining. As shown in Figures 4(b) and 4(d), there were more cells positively stained for VEGF in NGF-treated wounds than in saline-treated controls at 3 days and 7 days after wounding. However, the cells positive for VEGF were significantly downregulated at 14 days after wounding, while VEGF was undetectable in both NGF-treated and saline-treated wounds at 21 days after wounding. Thus, NGF might promote angiogenesis in cutaneous wounds by upregulating the expression of VEGF during the early stage of wound healing.

### 2.5. NGF Induced the Migration, but Not Proliferation, of Cultured Dermal Fibroblasts

**Figure 5:**
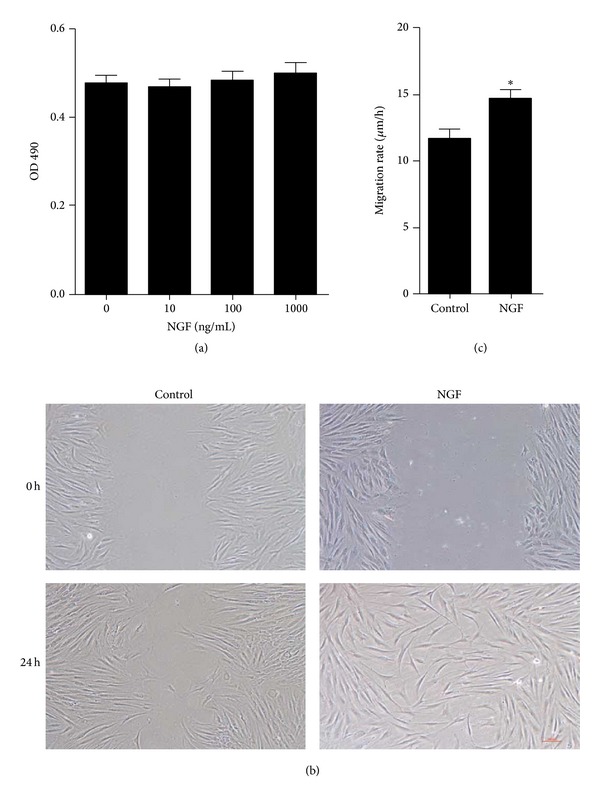
The effect of NGF on the proliferation and migration of human skin fibroblasts. (a) Cultured human dermal fibroblasts were incubated with different concentrations of NGF protein (0, 10, 100, and 1000 ng/mL) for 24 h, and cell proliferation was assessed by MTT assay. (b) Wound-healing assay of cultured human skin fibroblasts treated with saline or 100 ng/mL NGF for 24 h. (c) The migration rate of cultured fibroblasts after wounding is expressed as migration distance/time (*μ*m/h). *P < 0.05, compared to control group.

In light of the critical role fibroblasts play in cutaneous wound healing, we focused on the effect of NGF on cultured human dermal fibroblast. The effect of NGF on fibroblast proliferation was investigated with MTT assays. As shown in Figure 5(a), incubating the fibroblasts with different doses of NGF for 24 h did not promote fibroblast proliferation. In contrast, basic fibroblast growth factor (bFGF) could significantly induce fibroblast proliferation when incubated for 24 h (data not shown). However, fibroblast migration was remarkably induced by treatment with 100 ng/mL of NGF as compared to saline-treated control (Figures 5(b) and 5(c)). Thus, NGF plays a crucial role in fibroblast migration. The fibroblast migration enhanced by NGF may contribute to NGF-accelerated wound healing in excisional wounds in rat.

### 2.6. PI3K/Akt, Rac1, JNK, and ERK Were Involved in NGF-Promoted Fibroblast Migration

To clarify which signaling pathways are involved in the regulation of human fibroblast migration promoted by NGF, we further investigated the respective roles of PI3K/Akt, Rac1, JNK, and ERK in NGF-accelerated fibroblast migration.

**Figure 6:**
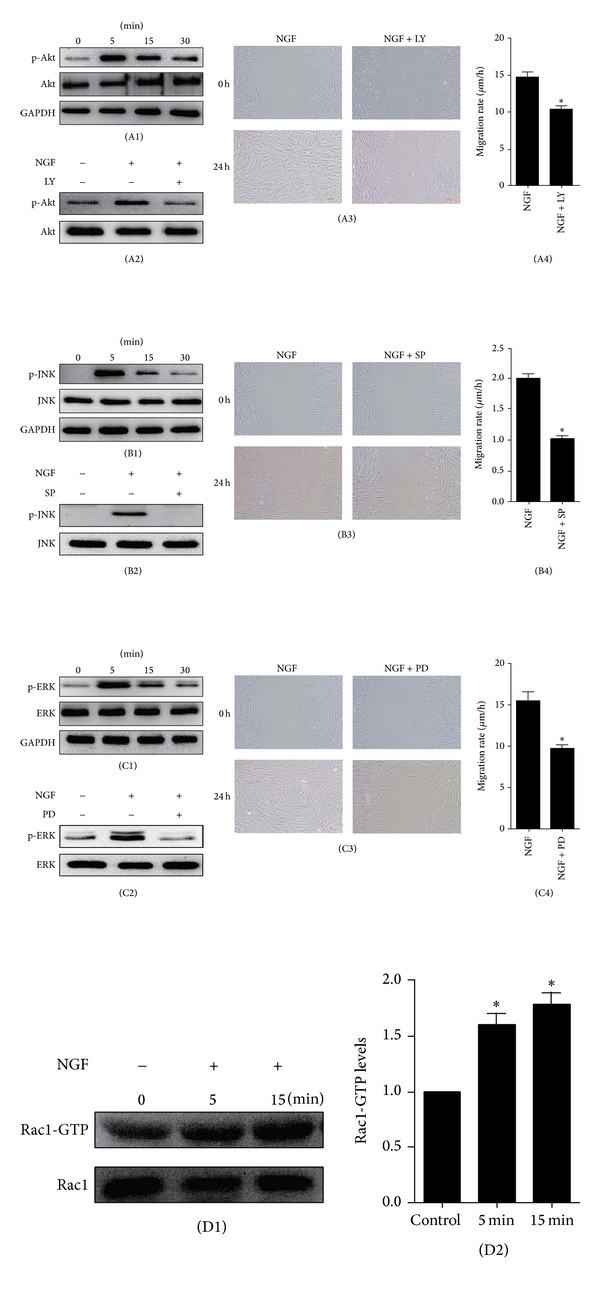
The effect on human skin fibroblast migration of blocking JNK, ERK, or PI3K/Akt pathways with specific inhibitors. ((A1), (B1), and (C1)) The levels of Akt, JNK, and ERK activity were all enhanced after treatment with 100 ng/mL of NGF for 5, 15, or 30 min. ((A2), (B2), and (C2)) The activity of Akt, JNK, or ERK following 5 min of NGF treatment was abolished by the corresponding specific inhibitor: LY294002 (10 *μ*M, LY is the abbreviation for LY294002), SP600125 (10 *μ*M, SP is the abbreviation for SP600125), or PD98059 (10 *μ*M, PD is the abbreviation for PD98059). ((A3), (A4), (B3), (B4), (C3), and (C4)) The NGF-induced migration of human skin fibroblasts induced by NG was significantly impaired after incubation with LY294002 (10 *μ*M), SP600125 (10 *μ*M), or PD98059 (10 *μ*M). *P < 0.05, compared to the control group. ((D1) and (D2)) Active Rac1 was pulled down and then detected by Western blotting. *P < 0.05, compared to untreated control group.

As shown in Figure 6(A1), 100 ng/mL NGF sharply raised the activity of Akt at 5, 15, and 30 min after treatment, though its peak effect was reached at 5 min. The NGF-induced increase in Akt phosphorylation in cultured human dermal fibroblasts was completely inhibited by LY294002, a specific inhibitor for Akt (Figure 6(A2)). When cells were pretreated with LY294002, NGF-induced fibroblast migration in the wound-healing assay was obviously impaired (Figures 6(A3) and 6(A4)).

The level of the phosphorylated JNK was significantly increased after stimulation with NGF (Figure 6(B1)). As shown in Figure 6(B2), when incubated with SP600125, a specific inhibitor for JNK, the NGF-induced phosphorylation of JNK in cultured fibroblasts, was completely blocked; thus the NGF-enhanced fibroblast migration was also significantly impaired in a wound-healing assay (Figure 6(B3)).

Similarly, ERK activity was significantly enhanced after treatment with 100 ng/mL of NGF (Figure 6(C1)). While the specific inhibitor for ERK, PD98059, markedly blocked the NGF-induced phosphorylation of ERK in cultured fibroblasts (Figure 6(C2)). When cells were pretreated with PD98059, NGF-induced fibroblast migration in the wound-healing assay was clearly reduced (Figure 6(C3)).

As shown in Figures 6(D1) and 6(D2), although total Rac1 content seemed unchanged after treatment with 100 ng/mL NGF, the level of active Rac1 was significantly increased over untreated controls as early as 5 min after NGF stimulation, and it remained high until 15 min. Thus, NGF can increase Rac1 activity, implying that Rac1 is probably involved in NGF-induced fibroblast migration. Taken together, these results suggest that PI3K/Akt, JNK, ERK, and Rac1 are all involved in the regulation of NGF-induced dermal fibroblast migration.

## 3. Discussion

Wound healing is an orderly and coordinated tissue repair process mainly consisting of three overlapping phases: inflammation, granulation tissue formation, and tissue remodeling. The healing process is highly regulated by cytokines, growth factors, and inflammatory mediators released from residential cells and infiltrating inflammatory cells in cutaneous wound tissue [1]. In intact skin, NGF is constitutively expressed by a variety of cell types, including fibroblasts and keratinocytes [13–15]. After skin wounding, the level of NGF is markedly upregulated, especially in the neonate [16]. The two specific receptors for NGF, p75NTR and TrkA, are expressed on the surfaces of various types of cells in the skin, including keratinocytes, melanocytes, fibroblasts, and mast cells [17–20]. The removal of the submaxillary glands, which store large amounts of NGF, substantially affects recovery after wound healing in mouse skin [8]. All of these findings suggest that NGF may play an important role in cutaneous biology and wound healing.

Indeed, it has been reported that topical application of NGF to cutaneous wounds hastened wound healing in normal and healing-impaired diabetic mice [7, 21]. A randomized clinical trial has also indicated that topical application of NGF could accelerate the wound-healing process in 18 selected patients with pressure ulcers of the foot [22]. We made a full-thickness excisional skin wound model in rats and topically applied different doses of NGF every other day to the wound. Our results showed that topical application of NGF with doses of 10 *μ*g or 20 *μ*g per wound also significantly accelerated the rate of wound healing in rats and enhanced wound reepithelialization and the formation of granulation tissue. Further increasing the dose to 40 *μ*g showed less of an effect on wound healing.

By immunohistochemical staining for PCNA, NGF obviously increased the cell proliferation in wound area. NGF significantly stimulates the proliferation of normal human keratinocytes in culture in a dose-dependent manner [13]. NGF has been shown to elicit broad biologic functions in inflammatory cells such as murine neutrophils and macrophages in the process of inflammation [23]. In agreement with this, we also found a significant increase in staining for CD68, a marker for macrophages, at the first week after NGF treatment, implying that NGF could recruit inflammatory cells including macrophages into the wound area at the inflammation stage of wound healing. There was also a mild increase in collagen content in healing wounds in the NGF-treated group compared to untreated controls, as demonstrated by Masson's trichrome staining. It has been reported that NGF does not influence the production of collagen in cultured fibroblasts, which is different from our finding [24]. This difference is probably attributable to the more sophisticated healing microenvironment* in vivo* involving the interaction of NGF with other growth factors and extracellular matrix molecules.

TGF-*β*1 has been extensively reported to elicit collagen synthesis in fibroblasts [25]; we measured the expression of TGF-*β*1 in wounds and found that the level of TGF-*β*1 was significantly increased in NGF-treated wounds compared with untreated controls. This implies that NGF may promote collagen production in wounds by upregulating the expression of TGF-beta. It has been suggested that supplementation of NGF promotes angiogenesis in mice with limb ischemia through a VEGF-dependent mechanism [26]. Consistent with this, we detected a significant increase in VEGF levels in wounds during the first week following NGF treatment. However, at 14 days after NGF treatment, the expression of VEGF in wounds was much lower than in untreated controls because the NGF-treated wounds were nearly closed. NGF did not promote the proliferation of cultured dermal fibroblasts derived from human foreskins, which is consistent with the findings of Micera et al. [24]. However, the migration ability of dermal fibroblasts was significantly enhanced following NGF treatment, suggesting that NGF-induced fibroblast migration may also contribute to NGF-accelerated skin wound healing* in vivo*. Our results, combined with others' reports, demonstrate that NGF influences wound-healing, probably by regulating inflammation and promoting angiogenesis and the proliferation of keratinocytes.

To clarify which signaling pathway is involved in the regulation of human fibroblast migration promoted by NGF, we first investigated the specific role of PI3K/Akt in NGF-accelerated fibroblast migration. The activation of the PI3K/Akt pathway plays a central role in establishing cell polarity and migration speed and is therefore required for the migration of various cell types, including fibroblasts [27–29]. NGF has also been shown to induce cell migration through the activation of PI3K/Akt pathway in rat peritoneal mast cells and aorta endothelial cells [30]. In our study, the cultured human skin fibroblasts showed an obvious increase in Akt activity after treatment with NGF. The inhibition of Akt phosphorylation by LY294002 significantly inhibited the migration of skin fibroblasts accelerated by NGF. This implies that PI3K/Akt signal transduction is involved in the regulation of NGF-promoted fibroblast migration. Accumulating evidence also suggests that the JNK pathway is important in regulating cell migration [31, 32]; by the same methods, the result is implying that the JNK signaling pathway also plays a crucial role in NGF-boosted fibroblast migration.

Rac1, a member of the Rho family of proteins, regulates actin organization and cell-cell adhesion and migration. Dominant-negative Rac1 inhibits lamellipodium extension, membrane ruffling, and migration in multiple cell types, including macrophages, T cells, epithelial cells, and fibroblasts [33]. Mutationally activated Rac1 potently and selectively activates JNK without affecting MAPK and dominant negative mutants of Rac1 block the JNK activation induced by cytokines and growth factors in COS-7 cells, suggesting that Rac1 plays a critical role in controlling the JNK signaling pathway [34]. PI3-kinase, Rac1, and JNK are all involved in bFGF-induced fibroblast migration and PI3-kinase is upstream of Rac1 and JNK is downstream of Rac1 [35]. Based on these findings, we next examined the effect of NGF on activation of Rac1. The activity of Rac1 was significantly increased after five minutes of NGF treatment. Taken together, our results suggested that PI3K/Akt, Rac1, and JNK are all involved in the regulation of NGF-induced dermal fibroblast migration and that it is mediated by a PI3K/Akt-Rac1-JNK signaling pathway.

Extracellular signal-regulated protein kinase (ERK) plays a crucial role in the regulation of cell survival, proliferation, and differentiation. ERK has also been reported to be involved in regulating cell migration, and the duration and magnitude of ERK activation associate with cell motility [36]. The inhibition of ERK activation results in markedly reduced movement of epithelial and endothelial cells in wound-healing experiments [37, 38]. Moreover, some reports have suggested that the ERK pathway is required for fibroblast migration and is potentially involved in determining movement direction [29, 39–41]. Previous studies in rat peritoneal mast cells and aorta endothelial cells also showed that NGF was able to induce directional cell migration through activation of the ERK pathway [30, 42]. Our study indicated that ERK is significantly activated following NGF treatment. The NGF-enhanced migration ability of fibroblasts was notably impaired when ERK activity was inhibited by PD98059, suggesting that ERK was also involved in the NGF-enhanced fibroblast migration. However, the detailed signal transduction cascade underlying NGF-induced fibroblast migration remains to be elucidated.

In conclusion, in this study, we provided experimental evidences that NGF administration may be used for the treatment of wound healing. NGF could speed up the reepithelialization, the formation of granulation tissue, and collagen production in cutaneous wound via regulating the expression of CD68, VEGF, PCNA, and TGF-*β*1. We also found that NGF could significantly promote the migration, but not proliferation, of dermal fibroblasts, which may contribute to the healing-promoting effect of NGF in skin wound. The activation of PI3K/Akt, Rac1, JNK, and ERK was all involved in the regulation of NGF-induced fibroblast migration. However, to apply NGF for the treatment of cutaneous wound in a clinical setting, a lot of work needs to be done. For example, the best administration dose, the interval of dosing, the therapeutic window of NGF, and its synergistic effect with other growth factors in wound healing remain to be further investigated in the future. Given the confirmative effect of NGF to promote cutaneous wound healing, further study to improve the pharmacodynamic action of NGF and to explore its underlying complicated mechanisms in wound healing is definitely warranted.

## 4. Materials and Methods

### 4.1. Ethics Statement

All experimental animals were provided by the Laboratory Animals Center of Wenzhou Medical University. The care and use of laboratory animals were strictly in accordance with international ethical guidelines and the National Institutes of Health Guide concerning the Care and Use of Laboratory Animals. The experimental procedures were carried out with the approval of the Animal Experimentation Ethics Committee of Wenzhou Medical University. Human foreskins were obtained from Second Affiliated Hospital of Wenzhou Medical University. Written consent was obtained from all participants involved in this study. The study protocol was approved by Institutional Ethics Committee of Second Affiliated Hospital of Wenzhou Medical University (Wenzhou, China) and informed consent was obtained from patients.

### 4.2. Culture of Human Skin Fibroblasts

Human foreskins were obtained from Second Affiliated Hospital of Wenzhou Medical University, and the primary culture of human dermal fibroblasts (HDFs) was established as described [43]. Briefly, the foreskins were washed three times in phosphate buffered saline (PBS) solution containing 1% penicillin/streptomycin sulfate. Subsequently, the tissues were digested with 0.5% dispase II overnight. The epidermis and subcutaneous tissue were excised from the tissues, which were then cut into pieces of approximately 1 × 1 × 0.5 cm and placed as explants in T25 tissue culture flasks. Growth medium was Dulbecco's modified Eagle's medium (DMEM) containing 5.5 mM D-glucose, 20% fetal bovine serum (FBS), 1% penicillin/streptomycin sulfate, and 2 mM L-glutamine. The primary fibroblasts were grown at 37°C in an atmosphere of 5% CO_2_ and were passaged every 2 days by trypsinization. Cells in passages three to six were used for all experiments in this study.

### 4.3. Preparation of Rat Cutaneous Wounds and Experimental Design

Male Sprague-Dawley rats, weighing 220–250 g, were chosen for this experiment. A wound healing model in rats was prepared as previously described [44]. Animals were anaesthetized by an intraperitoneal injection of 10% chloral hydrate (3.5 mL/kg) and positioned on a cork platform, and then hairs on the rat dorsum were clipped and then two 2 × 2 cm^2^ full-thickness skin flaps were cut per rat. The two wounds were separated by at least 1.5 cm of unwounded skin. The right-side (right) wounds were treated with different doses of NGF (Sigma-Aldrich, dissolved in 0.9% saline (w/v)), while the left-side wounds received equal volumes of 0.9% saline (w/v) as untreated saline controls. Wounded rats were randomly divided into three groups (fifteen rats per group): (1) rats treated with 10 *μ*g of NGF in right wounds; (2) rats treated with 20 *μ*g of NGF in right wounds; (3) rats treated with 40 *μ*g of NGF in right wounds. NGF treatments were repeated under anesthesia every other day for 14 days. Postoperative analgesics and antibiotics were not administered because these drugs may influence the healing process and thereby confound the interpretation of experimental data.

### 4.4. MTT Assay

Cultured dermal fibroblasts were seeded on 96-well plates (1 × 10^4^ cells/well) and treated with different concentrations of NGF (0, 10, 100, and 1000 ng/mL) for 24 h. Then, 20 *μ*L of MTT (3-(4,5-dimethylthiazol-2-yl)-2,5-diphe-nyltetrazolium bromide) (5 mg/mL in PBS) was added to each well for 4 h. Cells were washed with PBS (pH 7.4), and 150 *μ*L of DMSO was added to each well to solubilize the formed formazan crystals. Fluorescence intensity was measured at 570 nm.

### 4.5. Quantitative Assessment of Wound Healing

The size of the wounded area was determined at 2, 4, 6, 8, 10, 12, and 14 days after wounding. Transparent paper was placed over each wound, and the shape of the wound was drawn on the paper. The transparent paper was then superimposed on 1 mm^2^ graph paper for calculating the surface area of the wound. The percentage of wound closure was calculated using the following formula: wound closure rate = (area of original wound − area of actual wound)/area of original wound × 100% [45].

### 4.6. Wound-Healing Assay

Confluent primary fibroblasts were cultured in DMEM containing 0.5% FBS for 24 h and were then wounded with a linear scratch by a sterile pipette tip. Images of the wounded cell monolayers were taken using a microscope immediately and 24 h after wounding. To observe the effect of NGF on fibroblast migration, the cells were treated with saline or 100 ng/mL NGF just before wounding. To investigate the role of PI3-kinase, JNK, or ERK in fibroblast migration, the cells were incubated with 10 *μ*M LY294002, 10 *μ*M SP600125, or 10 *μ*M PD98059, respectively, for 60 min before the wounding. Twenty cells at the wounded area per each experiment were randomly selected 24 h after wounding, and the distance between the selected cells and wound edge was measured with Image J software. Migration rate was expressed as migration distance/time (mm/h).

### 4.7. Histological Examination

Wounded skin was excised, fixed in cold 4% paraformaldehyde overnight, and embedded in paraffin. Tissue sections (5 *μ*m thick) were stained with hematoxylin and eosin (H&E) for morphological assessment and with Masson's Trichrome staining for collagen analysis. The stained section slides were imaged at a magnification of 40 or 200x using a Nikon Eclipse E800 microscope.

### 4.8. Immunohistochemical Staining

Immunohistochemical staining for PCNA, CD68, TGF-*β*1, and VEGF was performed with specific antibodies for each protein (Santa Cruz Biotech, Santa Cruz, CA). Sections were dewaxed and hydrated; endogenous peroxidase was blocked with 3% hydrogen peroxide for 10 min; nonspecific binding was blocked with 1% BSA for 30 min. Sections were incubated with primary antibodies (at a dilution of 1 : 100) overnight at 4°C. Biotinylated secondary antibodies were then applied at 1 : 200 for 30 min, followed by incubation with horseradish peroxidase- (HRP-) streptavidin at 1 : 400 for 30 min. Color development was performed with DAB for 3 to 5 min for all samples, followed by hematoxylin counterstaining, dehydration, and coverslipping. The immunopositive cells in five fields per section were counted using Image-Pro Plus software (Nikon, Tokyo, Japan).

### 4.9. Rac1 Pull-Down Assay

Rac1-GTP assays were performed with the Active Rac1 Pull-Down and Detection Kit using the manufacturer's recommendations (Thermo Scientific). Briefly, the cells were scraped into 1X lysis buffer containing 25 mM Tris-HCl, pH 7.2, 5 mM MgCl_2_, 1% NP-40, 5% glycerol, 150 mM NaCl, and 1% protease inhibitor cocktail and centrifuged for 15 min at 16,000 ×g. The total of 700 *μ*L of cleared cell lysates was split into 20 *μ*g aliquots. As negative and positive controls for the pull-down, two of the aliquots were added to 5 *μ*L of 100 mM GDP or 10 mM GTP*γ*S, respectively, and incubated for 1 h at 4°C with gentle rocking. The beads were washed three times with lysis buffer and heated for 5 min at 100°C in reducing SDS-PAGE sample buffer and then analyzed for activated Rac1 by Western blotting with a monoclonal antibody for Rac1. Total Rac1 was determined to compare the level of the activated Rac1 across experimental conditions.

### 4.10. Western Blot

Western blotting was performed as follows. The cells were lysed, and the collected protein samples were denatured and separated on 10% polyacrylamide gels before being transferred to polyvinylidene difluoride membranes. The membranes were incubated in TBS containing 5% nonfat milk and 0.05% Tween-20 for 1 h at room temperature and blotted with the specified primary antibodies overnight at 4°C. The primary antibodies used were anti-phospho-Akt (1 : 300, Santa Cruz Biotech, Santa Cruz, CA), anti-Akt (1 : 300, Santa Cruz Biotech, Santa Cruz, CA), anti-phospho-JNK (1 : 1000, Cell Signaling Technology), anti-JNK (1 : 1000, Cell Signaling Technology), anti-phospho-ERK (1 : 300, Santa Cruz Biotech, Santa Cruz, CA), anti-ERK (1 : 300, Santa Cruz Biotech, Santa Cruz, CA), and anti-GAPDH (1 : 300, Santa Cruz Biotech, Santa Cruz, CA). The membranes were washed with TBST for 15 min the next day. Next, the membranes were incubated for 1 h with an anti-mouse or anti-rabbit HRP-linked secondary antibody (1 : 3000 dilution) and washed with TBST for 15 min. The signals were then detected using Western blotting detection reagent. The Western blot results were further analyzed using Image J software.

### 4.11. Statistical Analysis

Data are expressed as the mean ± SEM. Statistical significance was determined with Student's *t*-test when there were two experimental groups. For more than two groups, statistical evaluation of the data was performed using one-way analysis of variance (ANOVA) followed by Dunnett's post hoc test. For all tests, *P* < 0.05 was considered significant.
